# A narrative review of paracetamol‐induced hypotension: Keeping the patient safe

**DOI:** 10.1002/nop2.943

**Published:** 2021-06-08

**Authors:** Tricia L. Young

**Affiliations:** ^1^ Australia and Bairnsdale Regional Health Service University of New England Armadale VIC Australia

**Keywords:** ‘acetaminophen’ and ‘hypotension’ and related search combinations ‘paracetamol’, ‘fever’, ‘low blood pressure’, ‘propacetamol’, ‘sepsis’, and ‘shock’

## Abstract

**Aim:**

To understand the prevalence and epidemiology of paracetamol‐induced hypotension and clinical implications for contemporaneous practice.

**Design:**

Narrative review.

**Methods:**

In May and June 2020, an open‐date literature search of English publications indexed in ProQuest, PubMed, and EBSCO was conducted with the search terms ‘acetaminophen’ and ‘hypotension’ and related search combinations (‘paracetamol’, ‘propacetamol’, ‘low blood pressure’, ‘fever’, ‘sepsis’, and ‘shock’) to identify peer‐reviewed publications of blood pressure changes after paracetamol administration in humans.

**Results:**

A pattern of blood pressure reduction following the administration of paracetamol is demonstrated in the 27 studies included in this review. Haemodynamic intervention often followed persistent blood pressure reduction, and was greatest in febrile critically ill patients who received parenteral paracetamol.

## 1 INTRODUCTION

Nurses are required to escalate concerns about the quality and safety of care and are accountable for incidents that occur on their watch. Nurses have reported paracetamol‐induced hypotension, through monitoring and surveillance (Lee et al., 2018). Nevertheless, paracetamol continues to be marketed and prescribed as a presumed safe and harmless drug. This poses a risk that paracetamol will cause haemodynamic instability, which vastly goes undetected, even in high‐risk patient populations (Bae et al., 2017; Kang et al., 2018). The risk warnings of current paracetamol manufacturers are questionable, because they do not compel clinical governors to regulate the administration of paracetamol in the hospital environment. This narrative review combines the nurse's voice with the scientific evidence presented in the literature, which demonstrates that paracetamol‐induced hypotension is a real‐world problem and identifies high‐risk patients. Through narrative synthesis, we suggest methods of early detection and prevention of paracetamol‐induced hypotension, wherever paracetamol is prescribed in the hospital environment.

## 2 BACKGROUND

To treat fever or not remains a perennial debate (Mohr and Doerschuk, 2013). The benefits of reducing the oxygen and energy consumption induced by fever are contended by the theory that fever inhibits bacterial growth and activates physiological mechanisms that encourage pathogen clearance (Chiumello et al., 2017). Worldwide, paracetamol is used to treat fever and is ubiquitously administered in the hospital setting in multiple forms (Chiam et al., 2018). Moreover, paracetamol is the preferred non‐etiotropic antipyretic to manage fever in patients with the coronavirus disease (Day, 2020). Despite a growing body of evidence suggesting that paracetamol induces hypotension in febrile patients (Bae et al., 2017), there are few apparent warnings within the disclosures by the pharmaceutical manufacturers (Kelly et al., 2016; Maxwell et al., 2019). Hypotension is well detected in the critical care environment, where nursing surveillance and haemodynamic monitoring occur continuously (Bose et al., 2016; Pfrimmer et al., 2017). Outside of this environment, monitoring and surveillance occur less frequently (Peet et al., 2019). This increases the risk of delayed detection of paracetamol‐induced hypotension, because paracetamol is considered a harmless drug. Despite evidence on methods to treat paracetamol‐induced hypotension (Kang et al., 2018; Lee et al., 2018; Schell‐Chaple et al., 2017), there is a paucity of knowledge about who is most at risk of developing the condition and of ways to prevent it. This review critically appraises the current knowledge of paracetamol‐induced hypotension to identify the gap in clinical practice and is intended to guide good governance, which prioritizes patient safety (Dresser, 2012).

## 3 REVIEW METHODOLOGY

### 3.1 Design

This narrative review and synthesis is underpinned by a postpositivist paradigm, which explores the concept of paracetamol‐induced hypotension and clinical implications of contemporaneous practice.

### 3.2 Method

**FIGURE 1:**
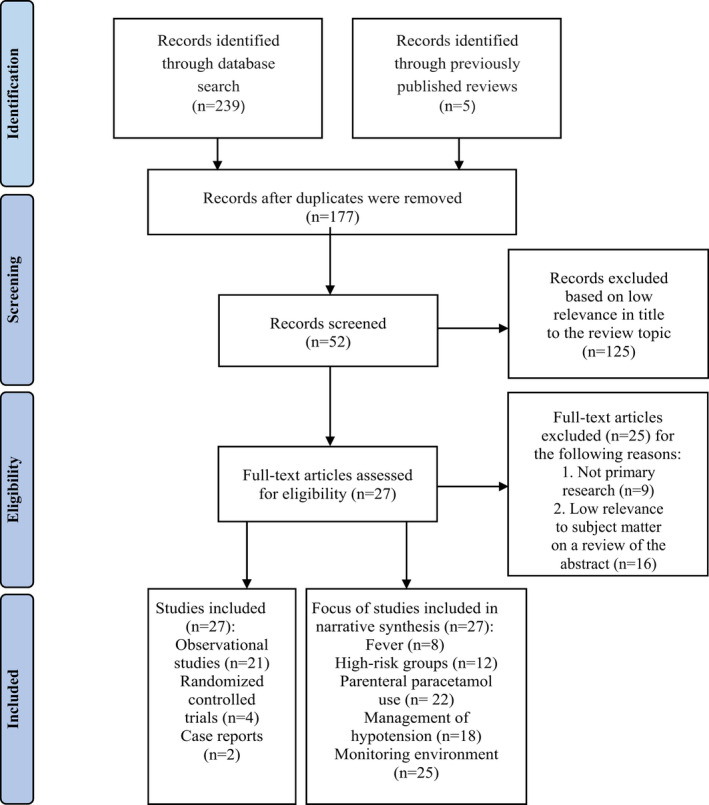
PRISMA search outcomes and records obtained from previously published reviews

A literature search was conducted between 18 May–30 June 2020 over the writing period of the review, to identify existing research that was commensurate with a broad area of interest: paracetamol‐induced hypotension (Figure 1). An open‐date search of medical subject heading keywords ‘acetaminophen’ and ‘hypotension’ were searched to yield articles indexed in PubMed, ProQuest and EBSCO databases. Multiple combinations of the related terms ‘paracetamol’, ‘propacetamol’, ‘low blood pressure’, ‘fever’, ‘sepsis’ and ‘shock’ were included in the search. The inclusion criteria were peer‐reviewed articles written in the English language and sorted in order of relevance. Two hundred and forty‐four articles were found. The publication types included primary and secondary journal articles, abstracts and reports. Literature reviews were manually searched for additional citations that were not extracted from the database search, whereas the reviews themselves were omitted. After duplicate articles were removed, 177 articles were identified. A further 125 studies were excluded if not primary research, grey literature, theses or if the title varied significantly from the haemodynamic effects of paracetamol, the key theme of this review. The abstracts of the remaining 52 articles were screened. Twenty‐five articles were then excluded when the abstract depicted low subject matter relevance. The remaining 27 articles were considered eligible, reviewed in full text and included in the study. The search was last updated on 30 June 2020. Herein, “paracetamol” refers to the active ingredient, acetaminophen, or propacetamol, the bio‐precursor (Duggan & Scott, 2009; Hersch et al., 2008).

### 3.3 Analysis

A narrative review and synthesis method was undertaken. This methodology enabled triangulation of concepts found within the literature to generate new knowledge and develop better ways to manage paracetamol administration in an acute‐care hospital setting. Contradictory research that refutes the paracetamol–hypotension association, the ways in which paracetamol‐induced hypotension is therapeutically managed, and the types of monitoring environment that can detect this condition are discussed.

## 4 RESULTS

In total, 27 articles were included in the final data set for review (Table 1).

**TABLE 1:** An overview of paracetamol‐induced haemodynamic changes

Author	Study design	Main study outcomes	Interventions		Setting
Brown (1996)	Two case reviews	Two patients consistently developed hypotension following paracetamol infusion administration (*N*= 2).	Both required vasopressors		ICU
Boyle et al., (1997)	Prospective observational	Paracetamol ↓SBP and ↓MAP in all recipients (*N* = 27). Paracetamol administration for febrile patients must be weighed against the possibility of causing a significant reduction in BP	29.6% required fluid bolus and/or vasopressors		ICU
Mackenzie et al., (2000)	Retrospective observational	IV paracetamol (*N* = 191) ↓BP ↓HR ↓BT within 2 hr following administration. Further research into the relationship between paracetamol and hypotension is warranted.	26.2% required IVF and/or vasopressors		ICU
Hersch et al., (2008)	Prospective observational	Propacetamol (*N* = 72) ↓BP ↓HR ↓BT at 15 min in febrile critically ill patients and induced clinically relevant hypotension necessitating intervention.	33% required IVF. 25% required IVF & vasopressors		ICU
Mrozek et al., (2009)	Prospective observational	IV paracetamol (*N* = 1507) ↓BP at 15–21 min in 1.33% of the sample. Hypotension occurred more frequently than specified in the drug information provided by the manufacturer. Acute brain injury and sepsis are risk factors.	0.66% required IVF and/or vasopressors		ICU
Allegaert and Naulaers (2010)	Retrospective observational	IV paracetamol (*N* = 72) ↓BP ↓HR 60 min after administration. Impaired haemodynamic status is suggested to be a relative contraindication to IV paracetamol use in neonates.	9% required unspecified therapeutic interventions		NICU
Boyle et al., (2010)	Prospective observational	IV or enteral paracetamol (*N* = 29) ↓BP in 59% of a febrile sample at 15–60 min. Paracetamol induces ↑skBF and may be associated with BP reduction in critically ill patients.	33% required unspecified treatment for hypotension		ICU
De Maat et al., (2010)	Retrospective observational	IV paracetamol (*N* = 38) ↓BP in 22% and 33% of patients at 15 and 30 min, respectively. The potential for IV paracetamol to cause hypotension in critically ill patients cannot be overlooked.	16% required intervention. 13% and 11% required IVF and vasopressors, respectively	ICU/MCU	
Duncan et al., (2012)	Prospective observational survey	IV or enteral paracetamol (*N* = 122) ↓SBP and ↓MAP with no change in HR or urinary output. Adverse effects of IV paracetamol may be underreported; 88% of nurses reported a preference for enteral over IV paracetamol due to the anticipated possibility of hypotension.	Not specified but ↓BP was reported in the whole sample		ICU
Krajcova et al., (2013)	Prospective observational	IV paracetamol (*N* = 48) ↓MAP by >15% in 42% of the sample. Mean onset time: 19 min. IV paracetamol led to hypotension in critically ill patients with cardiac insufficiency, thought to be caused by peripheral vasodilation in afebrile patients and a negative inotropic effect and ↓cardiac output in febrile patients.	42% required non‐specific therapy		ICU
Needleman (2013)	Retrospective observational	IV paracetamol (*N* = 100) ↓BP (SBP, MAP and DBP) at <5 min. No symptoms of hypotension were reported by patients. Rapidly infused paracetamol demonstrates no infusion‐related patient complications or side effects.	No intervention required to treat ↓BP		Preoperative
Picetti et al. (2014)	Prospective observational	IV paracetamol (*N* = 32) ↓BP ↓HR ↓CPP ↓BT at 30–120 min. Paracetamol is effective but exposes patients to hypotensive episodes that must be treated expeditiously to prevent further damage to the injured brain.	Norepinephrine infusions increased by 28%		Neurological ICU
Stoecker (2014)	Retrospective observational	IV paracetamol‐induced ↓BP in 90% of the sample (*N* = 393). A greater BP reduction was observed with the parenteral formulation than with enteral formulations. ICH patients have a higher incidence of acetaminophen‐induced‐hypotension.	Not reported		Surgical ICU
Chiam et al., (2015)	Blinded triple crossover RCT	IV paracetamol causes ↓BP immediately after infusion in healthy volunteers (*N* = 24); un‐associated with the mannitol constituent. The fall in BP is attributed to vasodilation. Supports changes in haemodynamic variables with IV paracetamol administration.	Not reported		Research laboratory
Cantais et al., (2016)	Prospective observational	IV paracetamol‐induced hypotension in 51.9% of the sample (*N* = 160) 30 min postinfusion. Adequately powered randomized controlled studies are recommended to provide confirmation, assess the physiologic mechanism involved and estimate the consequences.	34.9% of the patients required unspecified therapeutic intervention		ICU
Kelly et al., (2016)	Prospective, randomized, active‐control	IV and enteral paracetamol causes ↓BP (8.2%) at <60 min (*N* = 50). The incidence of paracetamol‐induced hypotension is higher than previously reported and is more frequent with the parenteral formulation.	69% required IVF and/or vasopressor use/dose increase		ICU
Yaman et al., (2016)	Single retrospective case report	Cardiac arrest precipitated by severe iatrogenic hypotension postparacetamol infusion (*N* = 1). Hypotension should be watched closely for after IV paracetamol administration.	Cardiac arrest, resuscitation, and postarrest inotrope administration		ED
Bae et al., (2017)	Retrospective observational	Propacetamol causes ↓BP ↓HR ↓BT at 30–60 min (*N* = 1507). Propacetamol can induce BP reduction in ED patients. This is greater in congestive heart failure patients and in those who experience chills prior to propacetamol administration.	10.7% required IVF and/or vasopressors		ED
Lee et al., (2017)	Retrospective observational	Of 4,771 hypotensive adverse drug reaction (ADR) events, 8.4% and 1.2% were recorded for propacetamol and paracetamol, respectively. Hypotensive events were commoner in older age groups and with concomitant drug therapy.	Not reported		Korean ADR database
Ray et al., (2017)	Prospective observational	In febrile children, IV or enteral paracetamol ↓BP (*N* = 148). Greatest BP reduction was at 2 hr postadministration. This effect was unrelated to BSA. The main effector was likely to be related to a change in the SVR.	22% required fluid bolus within 4 hr after dosing		Paediatric ICU
Schell‐Chaple et al., (2017)	RCT	IV paracetamol causes ↓BP, ↓HR, ↓BT <2 hr (*N* = 40). Further study of the antipyretic and haemodynamic response is warranted to inform evidence‐based practice guidelines for safe and effective fever management strategies.	35% required either IVF/up‐titration of vasopressors/ down‐titration of vasodilators		ICU
Chiam et al., (2018)	Single‐centre placebo RCT	25 cardiac surgery patients were randomized to IV paracetamol and 25 to saline. Thirty minutes postinfusion 14 hypotensive events occurred in the paracetamol group only.	8% sustained hypotension and received vasopressor therapy		Pre‐ and postoperative
Kang et al., (2018)	Retrospective observational	Propacetamol ↓BP (44.6%) ↓HR at ≤90 min (*N* = 195). Febrile UTI patients showed haemodynamic changes, although this did not affect the prognosis. In patients with normal BP or bacteraemia, the possibility of propacetamol‐induced hypotension should be considered.	11.79% required intervention; 11.2% required IVF; and 4.1% required vasopressors		ED
Lee et al., (2018)	Retrospective observational	Positive influenza A ED patients, postinfusion of propacetamol, showed ↓BP, ↓HR, and ↓BT compared with pre‐infusion values (*N* = 101). Significant BP reduction occurred in 29.7%.	Of those that experienced BP reduction, 20% required IVF		ED
Achuff et al., (2019)	Retrospective observational	20% had a 10% ↓BP from baseline and 5% had a 15% ↓BP from baseline at 60 min after IV paracetamol infusion (*N* = 777). Critically ill children with cardiac disease had a higher incidence of negative haemodynamic responses to IV acetaminophen.	16% required IVF ± Vasopressor support		PICU
Nahum et al., (2019)	Retrospective observational	IV paracetamol caused a haemodynamic event and ↓BP and ↓HR in 39% of patients after IV paracetamol administration (*N* = 100). The BP values reduced from high to normal for age and without any negative haemodynamic events.	No patient required additional vasopressor or IV therapy postdrug administration		PICU
Nahum et al., (2020)	Retrospective observational	IV paracetamol ↓MAP (*N* = 105) at 30, 60, 90 and 120 min after drug administration in 7.6%, 17.1%, 15.2% and 16.2% cases, respectively. Hypotensive episodes are common in critically ill children who receive IV paracetamol and those who are on inotropic support. Physicians should be aware of this potential risk, to ensure timely intervention.	11.4% of patients required a fluid bolus or vasopressor dose increase		PICU

Abbreviations: BSA, body surface area; BT, body temperature; CPP, cerebral perfusion pressure; DBP, diastolic BP; ED, Emergency Department; HR, heart rate; ICU, intensive care unit; IVF, intravascular filling; MAP, mean arterial pressure; MCU, medium care unit; NICU, Neonatal ICU; PICU, paediatric ICU; RCT, randomized controlled trial; sKBF, skin blood flow; SVR, systemic vascular resistance; SBP, systolic BP; UTI, urinary tract infection

### 4.1 Paracetamol‐induced hypotension: an overview

Brown (1996), in two retrospective case studies of febrile intensive care (ICU) patients, reported the earliest known evidence that paracetamol may induce hypotension based on nursing observation, which identified a distinct association between vasopressor up‐titration and paracetamol administration. Notably, a negative fluid balance was targeted in these patients to minimize the risk of pulmonary oedema (Brown, 1996). The author acknowledged the variations across ward and ICU monitoring environments, which accounted for the lack of evidence of paracetamol‐induced hypotension in general wards (Brown, 1996). Subsequently, a more representative retrospective chart review revealed the effects of paracetamol in 191 paracetamol administrations (Mackenzie et al., 2000). Baseline and hourly heart rate (HR), blood pressure (BP), and body temperature (BT) recordings were analysed up to three hours after paracetamol administration, and BT and BP reduction consistently occurred postdose. Noteworthy, intravascular filling (IVF) or vasopressor up‐titration occurred following 26.2% of paracetamol administrations. The authors suggested further research into paracetamol‐induced hypotension and highlighted the absence of warnings from paracetamol manufacturers that hypotension was a significant risk (Mackenzie et al., 2000).

Hersch et al., (2008) performed a prospective observational study that investigated the effect of parenteral propacetamol on the BP of critically ill febrile patients. Blood pressure, BT and HR were recorded before and at 15‐ and 30‐min intervals after propacetamol administration (72 administration‐related episodes occurred in 14 patients). The greatest BP reduction observed occurred 15 min postinfusion. In 33% of the cohort, corrective IVF was required to maintain haemodynamic stability, with 25% requiring up‐titration or initiation of vasopressor support. Besides propacetamol‐induced BP reduction, similar to early research (Boyle et al., 1997), the authors cautioned the need for an awareness of this potentially harmful effect among clinicians managing critically ill patients (Hersch et al., 2008). Cantais et al., (2016) conducted a similar prospective observational study of 160 ICU patients and reported that 51.9% of patients experienced BP reduction 30 min after paracetamol infusion, with more than a third of observed episodes requiring therapeutic intervention. The authors recommended adequately powered randomized controlled trials (RCT) to validate their findings (Cantais et al., 2016).

Boyle et al., (2010) performed another prospective study that explored the relationship between skin blood flow (skBF), paracetamol, and BP in critically ill febrile patients. The skBF was measured to compare with the haemodynamic responses after paracetamol administration in 29 febrile adults. The results showed that paracetamol induced a skBF increase and a corresponding BP reduction in 59% of the sample, of which 33% received treatment (Boyle et al., 2010) and the peak effect occurred at 60 min postadministration. Ray et al., (2017) performed a prospective observational study that further explored the paracetamol, BP and skBF association in 148 administrations of parenteral or enteral paracetamol in 31 children. The authors hypothesized that BP reductions would occur in febrile recipients and would be greater in children with a higher body surface area (BSA)‐to‐weight index. Significant BP reduction occurred after paracetamol administration, although this effect paradoxically did not occur in the higher BSA group (Ray et al., 2017). The authors concluded that although reductions in HR and stroke volume were contributing factors, the main effector of BP reduction was likely a reduced systemic vascular resistance and this effect was more significant in the sample that received parenteral paracetamol (Ray et al., 2017).

### 4.2 Patients suggested to be at greater risk of paracetamol‐induced hypotension

Potential associations have been previously indicated between paracetamol‐induced hypotension and febrile patients (Kang et al., 2018), with some patient groups being at higher risk than others (Bae et al., 2017; Picetti et al., 2014). Mrozek et al. (2009), in their prospective observational study, measured the incidence and pathophysiology of paracetamol‐induced hypotension and identified high‐risk patients in a cohort with 1,507 paracetamol administrations, regardless of the indication. Only 1.33% of their cohort experienced BP reduction, and half of those affected required therapeutic management. Among those who developed paracetamol‐induced hypotension, 80% were diagnosed with sepsis. The authors demonstrated that, statistically, patients with sepsis (*p* < .0001) and acute brain injury (*p* < .001) had a higher risk of developing paracetamol‐induced hypotension (Mrozek et al., 2009).

Lee et al., (2017) performed an even larger retrospective observational study, which explored the aetiology of hypotensive adverse drug reaction (ADR) events in a study population of 4,771 patients during a 4‐year period. Propacetamol was indicated as the likely precipitant for 8.4% of hypotensive ADRs, whereas paracetamol was the causative drug for 1.2% of reported drug‐associated hypotension. A significant proportion of the propacetamol‐ or paracetamol‐induced hypotensive episodes was reported as a serious event. Despite fewer paracetamol‐induced hypotensive events, both the seriousness thereof and the proclivity for this event in older patient populations were a remarkable association (Lee et al., 2017).

Picetti et al. (2014) performed a smaller prospective observational study in 32 patients in a neurosurgical ICU to explore the cerebral and haemodynamic effects of paracetamol in patients with acute brain injury, with hypovolemia as an exclusion criteria. The results showed that paracetamol‐induced reductions in temperature, BP and cerebral perfusion pressure. Due to the critical nature of hypotension in the study population, the proportion of patients who received vasopressor therapy increased from 47%–75%. The authors concluded that although paracetamol was effective in reducing potentially harmful fever in patients with acute brain injury, paracetamol confers a risk of hypotension, which requires judicious management to prevent further damage to the injured brain (Picetti et al., 2014). In another study, the incidence of hypotension following enteral or parenteral paracetamol administration in patients with cerebral haemorrhage was investigated (Stoecker, 2014). The authors reported a consistent incidence of hypotension within 60 min of paracetamol administration that was more pronounced in recipients of parenteral paracetamol. The author noted a greater prevalence of potentially harmful paracetamol‐induced BP changes existed in the clinical setting than was reported by paracetamol manufacturers (Stoecker, 2014).

In a retrospective study of 1,507 emergency department (ED) medical records, the temperature and haemodynamic values recorded pre‐ and postpropacetamol administration in febrile adult recipients were reviewed. Significant BP reduction occurred 30–60 min after propacetamol administration. In 10.7% of the participants, haemodynamic alterations that necessitated intervention occurred, all of which required IVF and 49% required vasopressor support. These results confirm that propacetamol provokes BP reduction in febrile patients and must therefore be carefully considered against the risk of causing haemodynamic alterations, in agreement with other research which demonstrates that paracetamol administration may not always be beneficial in febrile patients (Boyle et al., 2010). The clinical indicators that showed a greater tendency for propacetamol‐induced hypotension included concurrent heart failure or experiencing chills (Bae et al., 2017).

In another retrospective observational study of six critically ill patients who received paracetamol, 48 observation cycles of serial temperature and haemodynamic indices were reviewed (Krajcova et al., 2013). The authors reported paracetamol‐induced hypotension occurred in 45% of the sample. This effect was attributed to peripheral vasodilation and reduced cardiac output (Krajcova et al., 2013). Similar to previous research (Bae et al., 2017), the authors suggested that patients with cardiac compromise had a higher incidence of paracetamol‐induced hypotension. The findings of this study were supported by a single‐centre RCT by Chiam et al. (2018), who explored the haemodynamic effects of paracetamol versus normal saline in cardiac surgery patients. The authors concluded that pre‐operative paracetamol administration caused a transient BP reduction, but paracetamol can be safely administered without haemodynamic ramifications in the postoperative period. Of note, the paracetamol group received less IVF intraoperatively than their saline‐treated comparators, but received more IVF in the postoperative period, when the postoperative haemodynamic assumptions were made.

In a retrospective observational study, Allegaert and Naulaers (2010) determined that intravenous (IV) paracetamol should be a relative contraindication in neonates with impaired haemodynamic status. In a sample of 72 neonates, IV paracetamol‐induced mean arterial pressure and HR reduction, with 9% becoming hypotensive (Allegaert & Naulaers, 2010). A larger retrospective observational study exploring the haemodynamic effects of paracetamol indicated that critically ill paediatric populations with cardiac disease confounders were more susceptible to haemodynamic changes after IV paracetamol administration (Achuff et al., 2019).

A disturbing case report was published of a 2‐year‐old child who presented in a febrile and dehydrated state to an ED (Yaman et al., 2016). The child received IV paracetamol because of poor oral intake and subsequently experienced a catastrophic hypotensive episode that led to cardiac arrest. A retrospective review suggested that rapidly infused paracetamol and hypovolemia contributed to this sentinel event (Yaman et al., 2016).

### 4.3 Parenteral paracetamol‐induced hypotension

A number of studies have postulated a greater convergence between parenteral paracetamol and hypotension, as compared to the enteral form (Kang et al., 2018; Kelly et al., 2016; Nahum et al., 2019). A small‐scale prospective observational study by De Maat et al., (2010) reviewed the pharmacokinetic properties of paracetamol and compared serial BP responses following paracetamol administration in 38 patients in high‐dependency and ICU environments. Serial serum paracetamol, temperature and BP measurements were taken. Fifteen minutes postinfusion, 22% of patient's demonstrated BP reduction and this proportion increased to 33% at 30 min. At 60 min postinfusion, 16% required a corrective fluid bolus or vasopressors to stabilize the BP. The validity of the drug product information, which did not disclose the high incidence of hypotension, was once again questioned by the authors (de Maat et al., 2010).

Duncan et al., (2012) performed a retrospective observational study in the ICU setting to investigate the haemodynamic effect of centrally administered paracetamol to rationally determine the appropriate routes of paracetamol administration. Furthermore, nurses’ preferences of various paracetamol routes was surveyed, and 88% reported their preference for enteral paracetamol over parenteral formulations because of the anticipated hypotensive effects. The authors found that, in a sample of 122 paracetamol administrations, 17% of patient's demonstrated BP reduction, and all of them received the parenteral form. The authors theorized that an IV paracetamol‐induced BP reduction occurred and that the incidence of this phenomenon may be underreported (Duncan et al., 2012).

In contrast to previous observation studies, a non‐randomized retrospective study exploring the haemodynamic changes in suspected influenza A patients who received IV propacetamol was conducted by Lee et al., (2018). The inclusion criteria were normotensive, febrile, adult ED patients who had a positive result on a rapid influenza swab. Propacetamol induced a reduction in BP, HR and BT, and significant BP reduction was detected in 29.7% of patients, of whom 20% required IVF. Similar to Boyle et al., (2010), the authors suggested that a major change in skBF may have contributed to the observed propacetamol‐induced BP reduction (Lee et al., 2018). The study's retrospective nature was a limitation because BP monitoring was episodic and not protocol‐based. Therefore, the precise time at which the BP trough occurred may not have been recorded. Furthermore, the authors demonstrated that the pre‐drug BP values were higher in the group that experienced significant BP reduction and the postdrug BP values were similar. Nonetheless, the study supports the theory that IV propacetamol causes BP reduction in febrile patients.

A recent retrospective study by Kang et al., (2018) explored the haemodynamic responses of 195 febrile ED patients with urinary tract infection who received IV propacetamol. The authors acknowledged the existence of propacetamol‐induced hypotension and compared the clinical characteristics of patients who experienced persistent hypotension, determined by the need for IVF or vasopressor support for correction. The results revealed that the entire study cohort experienced a reduction in BP and HR, with 44.6% experiencing hypotension. Persistent hypotension occurred in 26% of patients and was more common in patients with a lower baseline BP or a higher risk of bacteraemia. The authors concluded that the significant incidence of propacetamol‐induced hypotension was not a detrimental prognostic indicator. Furthermore, the authors suggested that due to the likelihood of urogenic bacteraemia, these patients not only require appropriate monitoring but timely antibiotic and IVF administration (Kang et al., 2018).

Many researchers have used observational methods to validate the theory of paracetamol‐induced hypotension. Though inherently credible, these studies have been enhanced by the rigour of RCTs. Kelly et al., (2016) supported results in the prior literature in their prospective RCT, which explored the haemodynamic effects of parenteral‐versus‐enteral paracetamol administration in critically ill patients. Fifty febrile adult patients were randomly assigned in equal proportions to receive parenteral or enteral paracetamol for the treatment of pain or fever. Serum paracetamol concentrations and haemodynamic responses were monitored over 24 hr. In 12 patients, there were 16 hypotensive events (parenteral *N* = 12, enteral *N* = 4) within 60 min of paracetamol administration. Of those 16 episodes, 69% required discretionary rescue intervention with vasopressor up‐titration or IVF resuscitation. The authors concluded that the manifestation of paracetamol‐induced hypotension may be related to the ameliorating effects of treating pain or fever and the adjustment of the sympathetic tone. Similarly as in previous research, the authors suggested that parenteral paracetamol‐induced hypotension may be underreported by pharmaceutical manufacturers (Kelly et al., 2016). In another RCT, Schell‐Chaple et al., (2017) concluded that paracetamol modestly decreases BT and encourages clinically important BP reductions in febrile critically ill patients (Schell‐Chaple et al., 2017).

To stabilize the IV form of paracetamol, most of the commercially available preparations contain mannitol, which potentially has implications in haemodynamically vulnerable populations (Chiam, Weinberg et al., 2015). Following a review, a double‐blinded RCT was performed to compare the haemodynamic effect of paracetamol with saline and mannitol solutions in 24 healthy volunteers (Chiam, Weinberg, Bailey, et al., 2015). The results showed that paracetamol reduced BP and systemic vascular resistance immediately after the infusion was commenced, which was not demonstrated in the mannitol or saline comparators. This study was unique, because it demonstrated paracetamol‐induced BP reduction even in the absence of disease confounders. The authors attributed the BP reduction to paracetamol‐induced vasodilation (Chiam, Weinberg, Bailey, et al., 2015)).

More recently, Nahum et al., (2019) performed a retrospective chart review of 100 records to explore the haemodynamic effects of IV paracetamol in critically ill children. The results showed that 39% of children treated with paracetamol experienced a significant reduction in BP and HR within 120 min following the administration. The authors concluded that IV paracetamol provided pain relief and therefore enhanced a reductive effect on both HR and BP without haemodynamic compromise (Nahum et al., 2019). Following this chart review, Nahum et al., (2020) performed a retrospective observational study, which explored the haemodynamic effect of IV paracetamol administration in paediatric patients who were admitted to the critical care unit with septic shock and who also received inotropic support (Nahum et al., 2020). The study indicated that IV paracetamol administration caused significant mean arterial hypotension in 32.4% of recipients. The authors suggested that IV paracetamol administration in critically ill children in septic shock, who also require inotropic support, can exacerbate hypotension. The authors cautioned clinicians on the need for timely intervention of any resultant untoward haemodynamic manifestation (Nahum et al., 2020).

### 4.4 Contradictory research

In a retrospective study of 100 medical records from the perioperative environment, criticism of the haemodynamic safety of rapidly infused parenteral paracetamol was challenged (Needleman, 2013). The author concluded that although parenteral paracetamol‐induced BP reduction, contrary to previous studies, this was not considered clinically relevant (Needleman, 2013). There are a few reasons that these findings should be interpreted with caution and cannot be extrapolated to all patients. The sample frame was highly selective and limited to ambulatory preoperative patients, a perceivably healthy patient demographic. This prohibits the authors making a general recommendation, because it cannot draw comparisons with febrile critically ill patients and could be construed as optimistic bias (Jansen, 2016). The authors contended that BP reduction occurred postparacetamol administration because of a higher‐than‐normal pre‐treatment BP recording that was attributed to white coat syndrome, a hypothetical psychosomatic response in patients within hospital environments that transiently elevates their BP (Needleman, 2013). Furthermore, the study fails to explore the existence of haemodynamic change beyond 5 min of monitoring, suggesting that an element of knowledge distortion may co‐exist. Previous research has demonstrated that paracetamol‐induced hypotension occurs more than 15 min postinfusion and often persists for much longer (Kang et al., 2018; Lee et al., 2018). Moreover, a funding affect bias is possible given that the study's funding source was the same pharmaceutical company that produced the drug. It is important to bear in mind the potential for caveats of knowledge distortion in this study. Together, the methodological bias renders this knowledge doubtful and antithetically encourages hesitancy in clinicians when administering parenteral paracetamol.

## 5 DISCUSSION

### 5.1 Therapeutic management of hypotension

The prevalence of paracetamol‐induced BP reduction and the types of therapeutic intervention, if any, that was used to manage it has been summarized (Table 2). The adverse effects of sustained hypotension are well documented in the literature (Jones et al., 2006; Vincent & Leone, 2017) and are often a symptom that manifests in shock states (Shankar‐Hari et al., 2016), which includes heterogeneous causes in septic conditions, but may include vasoplegia (Sharawy, 2014) and a syndrome of fluid inadequacy and maldistribution (Shankar‐Hari et al., 2016). These tenets underpin research which suggests that early tailored IVF resuscitation and vasopressor therapy aim to optimize the haemodynamic conditions seen in septic shock states (Shankar‐Hari et al., 2016; Walker et al., 2014). Furthermore, Parkin and Leaning's mean systemic filling pressure concept suggests that intravascular space requires a level of static pressure to enable a viable haemodynamic state (Parkin, 2014; Parkin & Leaning, 2008). Despite many other variables to consider, the pragmatic inference suggests that, as the intravascular volume or circulatory fullness increases, so too does the mean systemic filling pressure and systemic venous return (Parkin, 2014; Parkin & Leaning, 2008). This research supports the theory that IVF is fundamental to maintaining a viable BP, which becomes critical in the management of hypotension in septic shock states (Beck et al., 2014). The significance of a greater hypotensive effect in heart failure (Bae et al., 2017) or in elderly patients (Lee et al., 2017) is important, because it conceivably relates to the iatrogenic minimalist approach to IVF. In heart‐failure or elderly patients, there is a tendency to impose fluid restrictions or only cautiously administer IVF for fear of overloading the diseased heart (Philipson et al., 2013). Integrating these concepts supposes that parenteral paracetamol has a greater hypotensive effect in patients with less intravascular reserve.

**TABLE 2:** Epidemiology of paracetamol‐induced blood pressure (BP) reduction

	Number of reported cases
Clinical setting	
Intensive care/High Dependency Units	19
Emergency department	4
Perioperative	2
General ward	0
Research laboratory	1
Not specified	1
Route of paracetamol administration	
Parenteral	25
Enteral	6
Peak time of BP reduction (min)	
≤5	2
6–15	3
16–30	5
31–60	9
61–120	3
Not reported	5
Reported intervention post‐BP reduction	
Intravascular filling	13
Vasopressors	16
Not reported	6
At‐risk diagnosis group identified	
Fever/sepsis	12
Paediatric/Neonatal	4
Elderly	1
Cardiac disease	4
Acute brain injury	3
Critically ill	19
Not specified	4

Vasopressor therapy is another strategy for managing hypotension in septic shock (Walker et al., 2014), despite the lower effectiveness in patients without sufficient intravascular volume. This focusses attention on IVF as the first‐line treatment of hypotension in septic shock (Walker et al., 2014). Thus, if an empty elastic tube (blood vessel) is compressed (vasopressor therapy), then the tube will essentially collapse. If the tube is prefilled before it is compressed, a turgor level is achieved within the vessel, enabling less need for vasopressor tension to achieve haemodynamic stability. Taken together, although these theories support the notion that optimal IVF prior to parenteral paracetamol administration in febrile patients may reduce the incidence of paracetamol‐induced hypotension, definitive evidence of this phenomenon is yet to be determined.

### 5.2 Monitoring environment

There is evidence that febrile critically ill patients are at the greatest risk of developing paracetamol‐induced hypotension, though this association is only made possible by the continuous monitoring environments these patients inhabit. Extensive research has been carried out on paracetamol‐induced hypotension in the critical care environment, whilst the availability of research pertaining to general ward environments is limited (Table 2).

**FIGURE 2:**
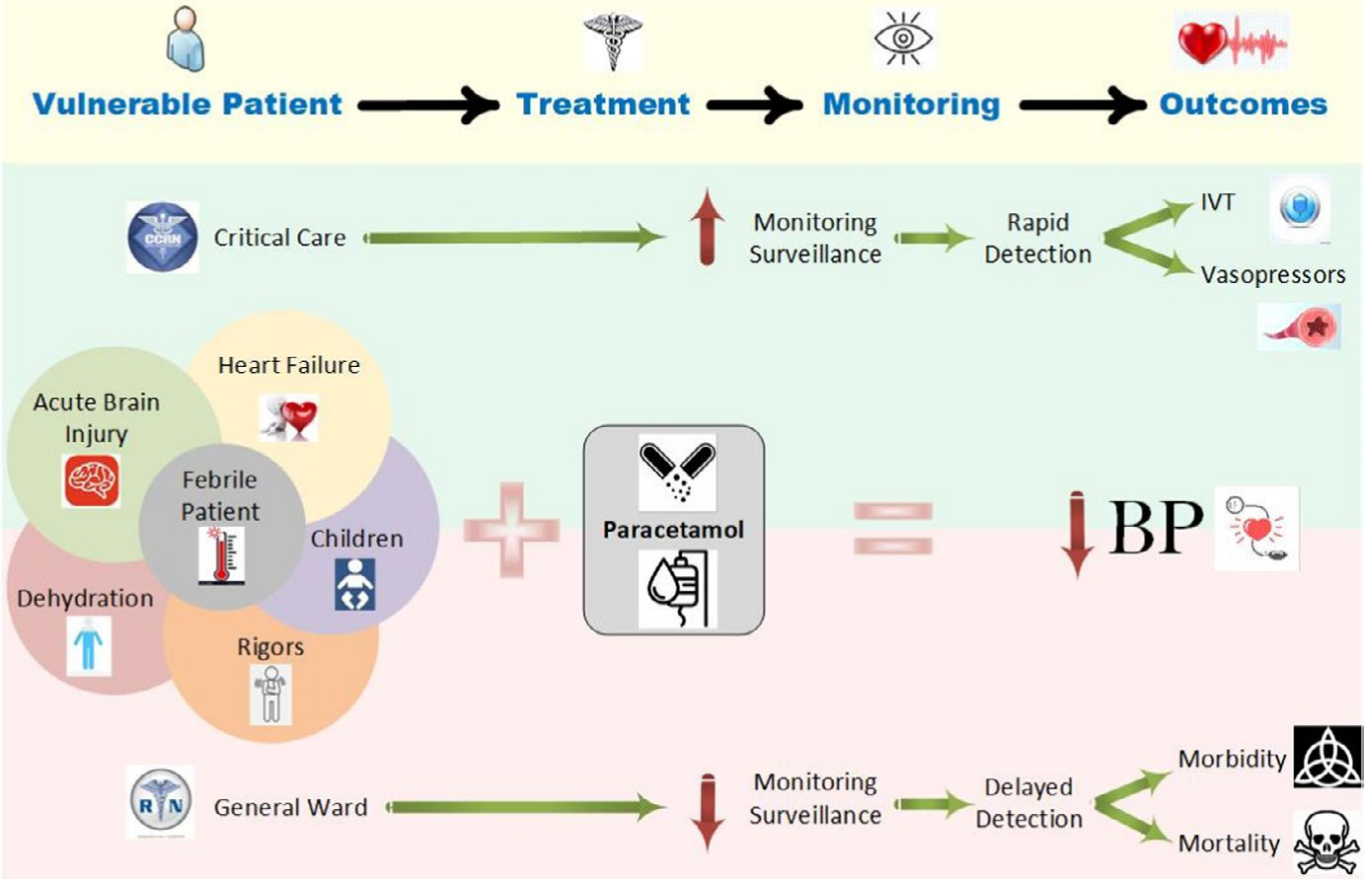
A concept map illustrating the hospital monitoring environment and paracetamol administration

Patients can and do deteriorate outside of critical care environments (Peet et al., 2019). Moreover, combining the variables of fever, hypovolaemia, and paracetamol prior to optimal fluid resuscitation poses a greater risk of hypotension. Capturing this effect is more likely to occur if BP monitoring is frequent, such as in higher‐acuity environments (Hope et al., 2018). Patients who inhabit the general ward environment, however, may be prescribed parenteral paracetamol for pain or fever, which is administered at the discretion of their bedside care providers. Without close observation, patients may have a paracetamol‐induced trough in BP, which vastly goes undetected (Figure 2). Despite the evidence described in the literature, there are currently insufficient haemodynamic warnings available within disclosures made by pharmaceutical companies that market paracetamol (Kelly et al., 2016; Maxwell et al., 2019). Counterintuitively, such omission can influence clinical practice guidelines for safe paracetamol administration.

### 5.3 Epidemiology

Taken together, the studies included in this review provide sound evidence that paracetamol can induce a prolonged episode of hypotension in febrile critically ill patients. This effect is perceivably more apparent in patient populations with less vasomotor control. Other significant epidemiological risk factors, which are consistent with this theory, include concurrent cardiac compromise and paediatric and elderly populations. The underlying aetiology and its extent are complex and can be influenced by pre‐existing conditions, age and the native influences of septic shock (Bae et al., 2017; Daniels et al., 2019; Ray et al., 2017). The heterogeneous nature of sepsis is a confounding variable, because patients are potentially already compromised by a hypovolaemic state (Semler & Rice, 2016) and vasoplegia (Sharawy, 2014). The syndrome of sepsis itself, though highly variable, requires a varied approach to fluid volume in resuscitation, which is often combined with vasopressor drug administration (Keijzers et al., 2020). Higher‐acuity monitoring and surveillance methods are required to detect instability in the haemodynamically vulnerable patient population to ensure early therapeutic management.

Unpacking this knowledge demonstrates that a corrective intervention is frequently required to maintain haemodynamic stability after the occurrence of paracetamol‐induced hypotension. Despite reports of how paracetamol‐induced hypotension is retrospectively managed, few insights have been suggested for prevention. In patients with an intrinsic tendency to develop paracetamol‐induced hypotension (Bae et al., 2017; Boyle et al., 2010; Picetti et al., 2014; Ray et al., 2017), it is worth considering whether parenteral paracetamol is safe to administer prior to achieving an optimal state of euvolaemia.

This evidence compels the arbitrators of safe governance to determine the appropriate hospital monitoring environment to detect paracetamol‐induced hypotension, which patients might receive paracetamol and the appropriate route of administration. The practice of close haemodynamic monitoring following paracetamol administration and correction of hypovolaemia prior to paracetamol administration should be of value to clinicians and should be widely applied in the hospital environment. This not only encourages practitioners to steward a safer and more rigorous monitoring strategy to detect paracetamol‐induced hypotension, but also surmises ways to prevent the condition.

### 5.4 Limitations

The outcomes of this review need to be considered in light of its limitations. The review makes recommendations for changes in governance and clinical practice, though the author did not observe actual clinical practice or consult directly with particular jurisdictions and organizations with regard to their local drug guidelines. The findings of this research may underestimate the degree of paracetamol‐induced hypotension, because no studies were performed outside of closely monitored environments. This limitation prevents comparisons of haemodynamic responses to paracetamol in less frequently monitored clinical environments. Furthermore, the majority of studies reviewed the haemodynamic effects of parenteral paracetamol, which prevents drawing accurate comparisons with the enteral form. The studies reviewed have used variable methods of BP monitoring. Furthermore, critically ill patients demonstrate differences in vasomotor tone and receive capricious amounts of IVF, which may influence the patients’ BP responses in each study. Moreover, it is feasible to suspect that a natural illness progression may result in hypotension, even in the absence of the drug. Though this review included RCTs, the majority of the sample comprised observational studies, which rely on the frequency of documented observations that predispose to inter‐study variations. Some of the publications included in this review contained only a limited documented critical appraisal, and the review itself does not contain a documented comprehensive critical appraisal of each study reviewed.

## 6 IMPLICATIONS OF THIS REVIEW FOR NURSING PRACTICE AND FUTURE RESEARCH

This review has important implications for future nursing practice in that paracetamol‐induced hypotension is evidenced in research and is better detected in close monitoring environments, to ensure timely intervention. There is scope for future research around paracetamol‐induced hypotension, which could include its occurrence in the general ward environment and a survey of existing acute hospital guidelines. Notably, the concept of pre‐emptive intravascular filling as a moderator variable emerges as a hypothesis, which could be usefully explored in future research.

## 7 CONCLUSION

The results of this review present a plethora of evidence that suggests a relationship between paracetamol administration and hypotension. Commensurate with the aims, this paper canvased epidemiological concepts and revealed populations that demonstrated a greater association between these variables. Furthermore, there is currently minimal contradictory research available that accurately contends the hypotension–paracetamol relationship. Despite the evidence, there is no effective system to regulate paracetamol administration, and the drug continues to be marketed without significant haemodynamic risk warnings. Overall, this review has important clinical implications for future practice and calls for a long‐awaited change in the overarching governance of this drug to protect the public and to minimize harm. This not only protects those receiving the drug, but also those administering it.

## CONFLICTS OF INTEREST

The author declares that there is no conflict of interest with respect to the research, authorship and/or publication of this review.

## ETHICAL APPROVAL

This review did not require research ethics committee approval.

## Data Availability

All data and evidence of this review are contained in the manuscript and are supplemented by figure 1 and tables 1 and 2, which were submitted as separate documents
